# From Plant By-Products to Insects to Shrimp: A Pathway to Sustainable Aquaculture Feed in a Circular Economy

**DOI:** 10.1155/anu/7288318

**Published:** 2025-06-26

**Authors:** Annalena Barth, Slim Bendag, Patrick Klüber, Daniel Kreft, Patrick Schubert, Dorothee Tegtmeier, Thomas Wilke

**Affiliations:** ^1^Institute for Animal Ecology and Systematics, Justus Liebig University Giessen, Heinrich-Buff-Ring 26, Giessen 35392, Germany; ^2^Branch for Bioresources, Fraunhofer Institute for Molecular Biology and Applied Ecology IME, Ohlebergsweg 12, Giessen 35392, Germany

## Abstract

Shrimp aquaculture often has a large environmental footprint, partly due to the fish meal content of commercial shrimp feeds. One potential way to improve ecological sustainability would be to replace fish meal-based compounds in shrimp feeds with insect-based compounds, such as black soldier fly (BSF; *Hermetia illucens* s.l.) larvae reared on plant by-products. However, little is known about the effects of novel plant by-products on the growth characteristics of BSF larvae and how these in turn affect aquaculture species, including Pacific white shrimp (PWS; *Penaeus vannamei*). Therefore, the general goal of this study was to evaluate the suitability of BSF larvae reared on three common plant by-products (cocoa bean shells, depectinized apple pomace, and potato peelings) as a feed component for PWS aquaculture based on randomized controlled feeding trials. The study showed that both the nutritional profiles of the tested feeds and the profiles of the BSF larvae reared on these feeds differed, particularly in crude protein and crude fat content. All BSF larvae reared on the plant by-products showed significantly lower growth performance than those reared on a control feed, possibly due to the presence of toxins and insecticides, and a low content of some essential amino acids. However, no significant differences in growth parameters were found between PWS fed BSF larvae reared on plant by-products and the control feed. Possibly, BSF do not bioaccumulate the toxins/insecticides present in the plant by-products and/or partially compensate for amino acid deficiencies. PWS fed BSF larvae reared on plant by-products had feed conversion ratios (FCRs) ranging from 0.8 to 1.0. These excellent values may fully or partially compensate for the lower growth performance of the BSF larvae reared on these by-products. This study may be of economic importance for future shrimp aquaculture, confirming that BSF can be a central link in the transformation of low-cost plant by-products into high-quality protein sources for sustainable shrimp farming in a circular economy.

## 1. Introduction

As oceans become increasingly overfished, marine aquaculture offers an alternative to fishing. However, many aquaculture practices, such as shrimp farming, are ecologically unsustainable [1]. In particular, the production of shrimp feed has a large environmental footprint. This is partly due to the fish meal content of commercial shrimp diets. Fish meal production is often associated with overfishing of fish species, the prevalence of foodborne marine pathogens, and high CO_2_ emissions associated with transportation [2]. Therefore, the development of responsible and environmentally friendly feed practices is key to sustainable shrimp aquaculture.

Scientists are, for example, working to replace fish meal-based compounds in shrimp diets with insect-based compounds such as fresh larvae or larval meal from the black soldier fly (BSF; *Hermetia illucens* s.l.) [3]. Many characteristics of the BSF, such as its rapid reproductive rate, low risk of disease transmission, and versatility in feed utilization [3, 4], high protein content [5], amino acid profile largely comparable to fish meal [6], potential for vertical farming [7], short production cycle, and possibility of local rearing [8], make it an attractive candidate for fish meal replacement. In addition, BSF contain antimicrobial peptides and chitin, which may induce beneficial immune responses in the target species [9].

However, the palatability of BSF compounds by shrimp may depend on the nutritional composition of the BSF larvae, including fat and protein contents, which in turn are influenced by factors such as developmental stage, rearing conditions, and feeding medium [10, 11].

Therefore, attempts are being made to use plant by-products as feed for BSF to reduce the fat content of the larvae while increasing the sustainability of insect rearing by exploring previously neglected by-products. In fact, large quantities of unused plant materials are generated as side streams in agriculture and the food industry [12]. These could potentially be used as sustainable feed for BSF rearing [13].

However, there are two critical research needs. First, researchers are interested in finding out how these novel plant-based feeds affect the developmental and growth characteristics of BSF, and second, how feeding BSF reared on plant by-products affects the developmental and growth characteristics of the aquaculture species such as shrimp.

Regarding the first point, studies have shown that the composition of the feed affects the growth and survival of BSF larvae [7, 13, 14]. For example, the use of apple pomace as insect feed resulted in reduced growth rates and an increased larval development time, probably due to the low protein, and high fiber content of apple pomace [7, 11, 15]. Similarly, feeding cocoa pods resulted in reduced growth and delayed development of BSF larvae [16]. In contrast, larvae reared on beer draff reached higher weights comparable to chicken starter mash [7].

Concerning the second point, there are only very few studies that have used BSF reared on plant by-products in shrimp feeding trials. For example, replacing 50% of the fish meal-based feed with bran-fed BSF had no negative effect on the growth performance or survival rate of shrimp [17]. Moreover, a mixture of bran and cereal meal as feed for BSF and the subsequent use of defatted BSF meal (30%–70% replacement of fish meal) led to improved growth of the shrimp compared to a fish meal-based diet [18]. Similarly, the use of food by-products as BSF feed and the replacement of up to 60% of the fish meal with the corresponding defatted BSF meal had no adverse effects on shrimp [19]. However, it remains unclear for most plant by-products to what extent they affect shrimp life-history parameters; and thus, shrimp aquaculture as a whole.

Worldwide, a large number of companies have emerged that produce the economically, most important shrimp species—the Pacific white shrimp (PWS; *Penaeus vannamei*)—locally in closed aquaculture facilities. They are increasingly interested in improving the sustainability of their operations by using shrimp feed with a lower environmental footprint. Therefore, the use of BSF reared on regional plant by-products could be an important step in this direction as part of a circular economy.

There are several plant by-products of low economic value that are available in large quantities worldwide and can be digested by BSF larvae directly or after moderate processing [10]. These include cocoa bean shells, depectinized apple pomace, and potato peelings.

Cocoa bean shells are a by-product of cocoa production. Cocoa pods are produced in vast quantities, especially in Africa, but also in the Americas and Asia. The beans are extracted, fermented, and dried. The dried beans are shipped around the world for, among others, chocolate production. During the process, the beans are deshelled, and the shells, which make up 10%–17% of the bean‘s weight, are mostly discarded. As a result, more than 700,000 tons of cocoa bean shells are generated each year, with Europe accounting for more than 250,000 tons [20].

Depectinized apple pomace is a by-product that remains after processing apples to remove pectin, a natural thickening agent used in various foods. The pomace typically contains the peels, seeds, cores, stalks, and remaining soft tissues of the apples. Worldwide, pectin production from apple pomace is an important industry, although exact production figures may vary. It is estimated that apple pomace accounts for a significant portion of the global pectin supply, with millions of tons of apple pomace processed annually [21].

Finally, potato peelings are a plant by-product of potato production and processing. Potatoes are one of the world's most important crops. During processing, 15%–40% of peel waste is generated. This results in at least 70,000–140,000 tons of potato peel by-products per year, most of which are disposed of in landfills [22].

Considering the great potential of neglected plant by-products in a circular economy, the general goal of this study is to evaluate the usability of BSF larvae reared on these by-products as sustainable feed for PWS aquaculture. Based on randomized controlled feeding trials, we specifically:

1. determined the nutritional profiles of three candidate- plant by-products—cocoa bean shells, depectinized apple pomace, and potato peelings—and compared them with a grain-based control feed,
2. evaluated the nutritional profiles of BSF larvae reared on these plant by-products compared to a control feed,
3. analyzed the growth performance of BSF larvae reared on these plant by-products compared to a control feed,
4. determined the growth performance characteristics of the PWS fed with BSF larvae reared on these plant by-products compared to a control feed, and
5. calculated the respective feed conversion parameters.

## 2. Materials and Methods

### 2.1. Ethics Statement

In Hesse, Germany, experiments with shrimps (order Decapoda) and flies (order Diptera) do not require a permit. All applicable legal requirements were met.

### 2.2. Species Studied and Pretreatment

Black soldier flies (BSFs) were obtained from Bio.S Biogas (Grimma, Germany) in July 2018 (Table 1). Since then, the population has been kept genetically isolated from other populations (>37 generations). Adult BSF flies were maintained in 60 cm × 60 cm × 90 cm (length × width × height) mesh cages (Bioform, Nürnberg, Germany), located in a greenhouse (Fraunhofer IME, Giessen) at 26 ± 1°C, 60% ± 5% relative humidity, and a 12 h photoperiod (L-PL-ECO623330, Lence Technology, Langen, Germany). Water-soaked paper towels provided drinking water ad libitum, and the mesh cages were sprayed with water daily. Fresh egg clutches were collected from artificial oviposition sites consisting of three wooden boards spaced by washers and held in place with rubber bands [23]. After weighing (ALJ 160-4A, Kern & Sohn, Balingen, Germany), 150 mg of eggs (approximately 6000 eggs) were transferred into 19.5 × 16.5 × 9.5 cm plastic boxes and moistened. Once ≥50% of the eggs had hatched, the neonates were initially fed 10 g of the corresponding feed. The boxes were monitored daily for remaining water and feed, and additional feed was provided ad libitum (typically at 48-h intervals). The moisture content of the diets was measured using a TMT-MC-7828S soil moisture meter (OCS.tec, Neuching, Germany) and adjusted to approximately 70% by spraying. Larvae were reared in a climate chamber at 27 ± 1°C and 65% ± 5% relative humidity in darkness [13].

Juvenile PWS (Table 1) were obtained from Suburban Seafood Germany UG (Nebelschütz, Germany). They were acclimated for 1 week in a wooden tank (Douglas fir, 1200 mm height, 600 mm radius, Wilhelm Eder GmbH, Bad Dürkheim, Germany), located at the marine aquaculture experimental facility of the Justus Liebig University Giessen, at a stocking density of 0.8 larvae L^−1^. The indoor recirculation system was equipped with a filtration system consisting of a drum filter (35 μm drum mesh, FAIVRE, Baume-les-Dames, France) and a protein skimmer (Bubble King DeLuxe 650 intern, Royal Exclusiv, Wesseling, Germany). The shrimp were fed six times per day ad libitum with the PWS compound feed Grower 1 (diameter 1.7 mm, Le Gouessant Aquaculture, Lamballe, France) through automatic feeders (easyFuttermat, Aqualight GmbH, Bramsche, Germany).

DNA barcoding of the mitochondrial cytochrome *c* oxidase subunit I (COI) gene in two individuals each of BSF and PWS was performed according to Barth et al. [6].

### 2.3. Nutritional Analyses of BSF Feeds and BSF Larvae

For the nutritional analyses, BSF larvae and the BSF feeds were lyophilized for 72 h, and then ground in a mortar. Prior to lyophilization, the moisture content was determined thermogravimetrically using an M35 moisture analyzer (Sartorius, Göttingen, Germany). For crude ash determination, the diets were pre-ashed in a quartz crucible over a Bunsen burner, incinerated twice at 550°C for 6 h in a muffle furnace (L 9/11, Nabertherm, Lilienthal, Germany), and the content calculated by differential weighing. The total nitrogen content was determined by the Kjeldahl method [24]. Samples were subjected to sulfuric acid digestion (Kjeldatherm, C. Gerhardt GmbH & Co. KG, Königswinter, Germany), followed by automated steam distillation and titration using the Vapodest 500 (Vapodest 500, Gerhardt, Königswinter, Germany) and TitroLine 5000 (SI Analytics, Mainz, Germany) systems. A conversion factor of 6.25 was used to calculate the crude protein content [25]. For the determination of crude fat content according to Weibull-Stoldt, samples were first manually disintegrated in 150 mL of boiling 4 mol·L^−1^ HCl for 30 min. They were then filtered, washed neutrally with hot demineralized water, and dried at 105°C for 2 h. Crude fat extraction was performed automatically with n-hexane in a Soxtherm system (Gerhardt, Königswinter, Germany). The content was determined gravimetrically.

### 2.4. Plant By-Products and Control Diet Used as BSF Feeds

Four diet groups were established for the feeding trials: cocoa bean shells, depectinized apple pomace, potato peelings, and a grain-based control feed. Cocoa bean shells were provided by August Storck KG (Berlin, Germany). The depectinized apple pomace was obtained from agro Food Solution GmbH (Herbavital F12, Werder, Germany). Both cocoa bean shells and depectinized apple pomace were homogenized in a Thermomix TM6-1 for 1 min at setting 10 (Vorwerk, Wuppertal, Germany). The control feed was ground in a Mockmill 200 grain mill (Wolfgang Mock, Otzberg, Germany) and served as reference feed (GoldDott Eierglück, Derby Spezialfutter, Muenster, Germany). After processing, control feed, cocoa bean shells, and depectinized apple pomace had a particle size of 0.1–1.5 mm, which was determined with analytical sieves (Retsch, Haan, Germany). Due to their low moisture content (6.5%–9.5%), the feed was stored at room temperature in separate airtight plastic barrels until further use.

Potato peelings were produced by peeling potatoes (variety “Kuras”) on a Flott 16K potato-peeling machine (Flottwerk, Rotenburg a.d. Fulda, Germany) and were supplied frozen by the Fraunhofer Institute for Process Engineering and Packaging in Freising (Germany). Due to the high moisture content (82%), the potato peelings were stored at –20°C and processed directly on the day of feeding. The potato peelings were mixed into a slurry using the Thermomix TM61 for 10 min at setting 6–8. Excess water was drained before feeding. In addition, part of the potato peelings was dried for 12 h at 50°C (BDA-15 dehydrator, Beeketal Lebensmitteltechnik, Rastdorf, Germany) and ground to adjust the feed moisture during feeding.

### 2.5. BSF Feeding Trials

For each BSF diet group, developmental and growth parameters were recorded using three replicate boxes (Table 2). The boxes were maintained under the conditions described above. Growth parameter documentation was started when the larvae reached a manageable size of 3–4 mm, which was highly dependent on the feed. To obtain growth curves, the mean weight of 25 randomly selected larvae was determined using an AT261 DeltaRange analytical balance (Mettler, Giessen, Germany). Measurements were stopped and boxes were harvested when ≥50% of the population had reached the prepupal stage, indicated by brown coloration. The larvae were separated from the substrate by sieving (AS 200, Retsch, Haan, Germany), cleaned of impurities, and stored at –20°C for subsequent analysis and feeding to shrimp.

### 2.6. PWS Feeding Trials

For the PWS feeding trials (Table 2), a combined diet was used. It consisted, on a dry matter weight basis, of approximately 50% of the conventional fish meal-based compound feed Grower 1 and approximately 50% BSF larvae. This ratio has been suggested to have no adverse effects on the growth performance and survival rate of PWS [17]. The BSF larvae were reared on cocoa bean shells, depectinized apple pomace, potato peelings, or a control feed. For the latter, we used a grain-based diet, as this is a common feed for BSF larvae [7]. The relative amount of feed supplied daily was approximately 1.5% compound feed and approximately 1.5% BSF larvae of the PWS biomass on a dry matter basis (for total amounts of feed supplied, see https://doi.org/10.22029/jlupub-19060). An automatic feeder was used to deliver the compound feed to the PWS. The BSF larvae were fed manually after thawing and cutting into pieces of 2–3 mm. For the calculation of feed conversion ratios (Section 2.7, Equation (2)), it was assumed that all the feed was consumed by the shrimp.

The PWS were preselected for the trials based on weight and length criteria (Table 1), and randomly distributed to the treatment and control tanks. Shrimp were kept individually to prevent competition and cannibalism. Each plastic tank had a clear front screen, blue-opaque side walls, and an acrylic-glass lid to prevent shrimp escape and to reduce evaporation. Residual feed, shells, and feces were siphoned off once a week. For a detailed description of the “single-shrimp system” used, see [26].

Tank water parameters (Table 3), including nitrogen, nitrate (test strips QUANTOFIX Peroxid 25, Macherey-Nagel, Düren, Germany), salinity, and temperature (Hanna Instruments Deutschland, Vöhringen, Germany), were monitored daily. Dissolved oxygen and pH were measured twice a week with a multiparameter probe (Multi 3620 IDS SET G, Xylem Analytics Germany, Weilheim, Germany). All information provided for PWS (Tables 1–3) complies with the guidelines for reporting experimental studies in shrimp [6].

### 2.7. Data Processing and Statistical Analyses

Our randomized controlled trials followed the Consolidated Standards of Reporting Trials (CONSORT) guidelines [27]. Statistical analyses of data were performed in Excel 2016 (Microsoft, Redmond, USA) and OriginPro 2022b (OriginLab, Northampton, USA). With a coefficient of determination of *R*^2^ = 0.26, a statistical power of 0.85, and a significance level of *α* = 0.05, a sample size of *n* = 28 would be required for a significant overall model with one predictor [28]. These requirements were met for both the BSF and PWS feeding trials. Shrimp survival was analyzed using the nonparametric Kaplan–Meier estimator. The *S* (*t*) survival functions were then compared pairwise using log-rank tests (*α* = 0.05). The homogeneity of variance was assessed by Levene's test. For all other parameters, a one-way analysis of variance (ANOVA) was performed, and mean values were separated using Tukey's test (homogeneous variance). When the assumption of homogeneity of variance was not met, we used Welch's one-way ANOVA, followed by a Games–Howell post hoc test [29]. Linear relationships were calculated using Pearson product–moment correlation [30]. Visualization of the data was conducted in the R statistical environment, version 4.3.1, together with the graphical user interface 'RStudio' (version 2023.06.1 + 524, Posit PBC, Boston, USA).

The following formulas were used to obtain the efficiency of conversion of ingested feed (ECI; Equation (1)), the feed conversion ratio (FCR; Equation (2)), the specific growth rate (Equation (3)), the survival rate (Equation (4)), and the weight gain (Equation (5)); (DM, dry matter; FM, fresh matter):

(1) $$\begin{array}{c} \text{Efficiency of conversion of ingested feed }=\frac{\text{total biomass harvested }\left(g,\text{ FM}\right)-\text{initial biomass }\left(g,\text{ FM}\right)}{\text{total feed provided }\left(g,\text{ DM}\right)}, \end{array}$$

(2) $$\begin{array}{c} \text{Feed conversion ratio}=\frac{\text{total feed intake }\left(g,\text{ DM}\right)}{\text{final body weight }\left(g,\text{ FM}\right)-\text{initial body weight }\left(g,\text{ FM}\right)}, \end{array}$$

(3) $$\begin{array}{c} \text{Specific growth rate }\left(\text{\% per day}\right)=\frac{100\times \left(\text{Ln final body weight}-\text{Ln initial body weight}\right)}{t\left(\text{days}\right)}, \end{array}$$

(4) $$\begin{array}{c} \text{Survival rate }\left(\text{\% }\right)=\frac{100\;\times \text{final number of shrimp}}{\text{initial number of shrimp }}, \end{array}$$

(5) $$\begin{array}{c} \text{Weight gain }\left(\text{\% }\right)=\frac{100\times \left(\text{final body weight}-\text{initial body weight}\right)}{\text{initial body weight}}. \end{array}$$

## 3. Results

### 3.1. Nutritional Profiles of Plant By-Products and Control Feed

Information on the nutritional profiles of the three plant by-products tested (i.e., cocoa bean shells, depectinized apple pomace, and potato peelings) compared to a grain-based control feed is provided in Table 4. The three plant by-products differed from the control feed in some or all of the nutritional parameters analyzed. In particular, potato peelings contained up to 13 times more water than the control feed and the other diets (*Welch's F*_(3, 3.35)_ = 250.77; *p* < 0.001). The crude ash content of the control feed was significantly higher than that of any of the plant by-products tested (*Welch's F*_ (3, 4.07)_ = 1121.34; *p* < 0.05). Cocoa bean shells had the highest crude fat content, exceeding the control feed and the other diets by at least 940% (*Welch's F*_ (3, 4.19)_ = 1792.14; *p* < 0.001). The crude protein content of apple pomace and potato peelings was significantly lower than in the control feed and in cocoa bean shells, as was the total nitrogen content (*Welch's F*_ (3, 3.88)_ = 9550.25; *p* < 0.001).

### 3.2. Nutritional Profiles of BSF Larvae Fed With Plant By-Products or the Control Feed

Information on the nutritional profiles of BSF larvae fed the three tested plant by-products (i.e., cocoa bean shells, depectinized apple pomace, and potato peelings) compared to a grain-based control feed is presented in Table 5. BSF larvae fed apple pomace and potato peelings had a significantly higher moisture content than those fed cocoa bean shells and the control diet (*Welch's F*_ (3, 3.39)_ = 4197.30; *p* < 0.05). The crude ash content of BSF larvae fed potato peelings or apple pomace was also significantly higher than that of those fed cocoa bean shells or the control feed (*F*_ (3, 11)_ = 36.40; *p* < 0.001). The fat content of the larvae varied greatly depending on the diet. Crude fat content was particularly low in BSF larvae fed apple pomace and potato peelings, compared to cocoa bean shells and the control feed (*F*_ (3, 11)_ = 755.02; *p* < 0.001). Both the total nitrogen and crude protein content of BSF larvae fed the three plant by-products were higher than of the larvae fed the control feed (*F*_ (3, 11)_ = 93.52; *p* < 0.001).

### 3.3. Growth Performance of BSF Larvae Fed With Plant By-Products or the Control Feed

**Figure 1:**
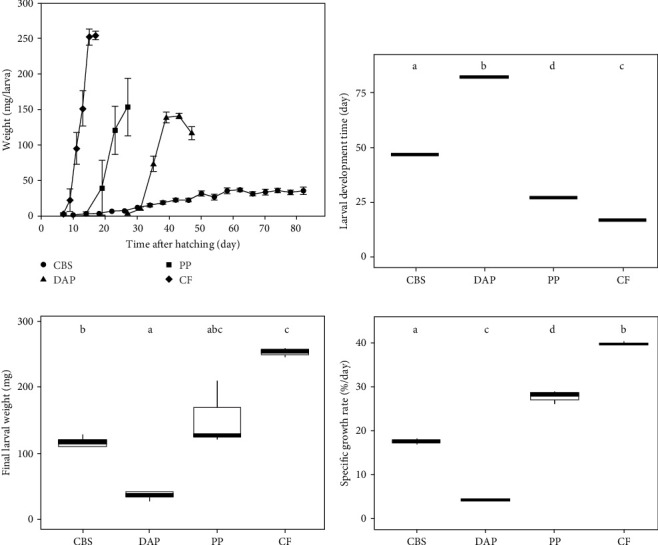
Growth curves (A), larval development time (B), final larval weight (C), and specific growth rate (D) of BSF larvae reared on three plant by-products (cocoa bean shells, CBS; depectinized apple pomace, DAP; potato peelings, PP) and a grain-based control feed (CF). Different letters (a–d) indicate statistically significant differences (*p* < 0.05; one-way ANOVA or Welch's ANOVA). Data for (B–D) are presented as box-and-whisker plots. Lines indicate medians and boxes first and third quartiles.

Information on the growth characteristics of BSF larvae fed the three plant by-products (i.e., cocoa bean shells, depectinized apple pomace, and potato peelings) compared to a grain-based control feed is summarized in Figure 1.

All BSF larvae fed plant by-products had a significantly longer development time compared to those fed the control feed (*F*_ (3, 10)_ = 9.58; *p* < 0.001) (Figure 1B). The development time was longest in larvae fed depectinized apple pomace, followed by those fed cocoa bean shells and potato peelings. Neither crude protein (*r* = –0.47; *p* = 0.08) nor crude fat (*r* = 0.00; *p* = 0.93) content of the feeds was correlated with larval development time.

BSF larvae fed the control feed exhibited the highest and fastest weight gain, followed by those fed potato peelings, cocoa bean shells, and depectinized apple pomace (Figure 1A). Accordingly, final larval weight differed significantly between groups (*Welch's F*_ (3, 4.16)_ = 504.61; *p* < 0.001), with the heaviest larvae reared on the control feed, followed by those reared on potato peelings, cocoa bean shells, and depectinized apple pomace (Figure 1C). Final larval weight was positively correlated with crude protein content of the diets (*r* = 0.54; *p* < 0.05), but not with crude fat content (*r* = –0.09; *p* = 0.76). The specific growth rate of BSF larvae differed significantly among diets, with all plant by-products showing lower rates than the control feed (*Welch's F*_ (3, 2.67)_ = 1587.68; *p* < 0.01) (Figure 1D).

### 3.4. Growth Performance of PWS Reared on BSF-Based Feeds

**Figure 2:**
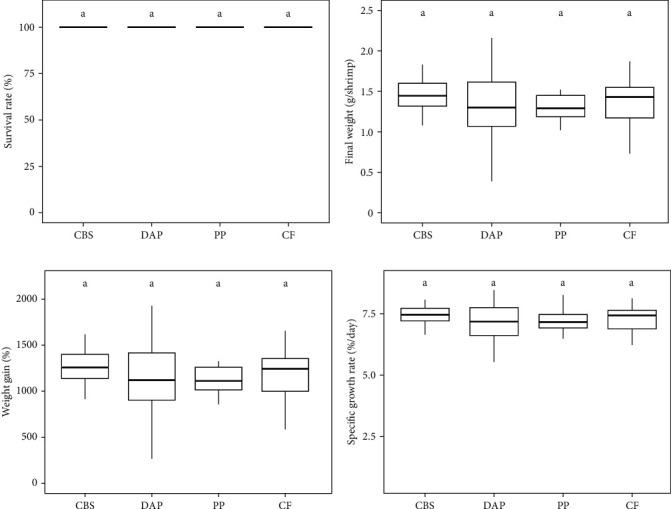
Growth characteristics of PWS ((A) survival rate, (B) final weight, (C) weight gain, and (D) specific growth rate) fed a diet containing BSF larvae reared on three plant by-products (cocoa bean shells, CBS; depectinized apple pomace, DAP; potato peelings, PP) or a grain-based control feed (CF). The letters “a” indicate that there were no significant differences between treatment and control groups (*p* < 0.05; KaplanMeier [survival], one-way ANOVA or Welch's ANOVA). Data are presented as box-and-whisker plots. Lines indicate medians and boxes first and third quartiles.

Information on the growth characteristics of the PWS at the end of the 35-day controlled trial is summarized in Figure 2. In the randomized trial, four diets were used for the PWS, which contained BSF larvae raised on plant by-products (i.e., cocoa bean shells, depectinized apple pomace, and potato peelings) or a grain-based control feed.

Shrimp survival rate was found to be consistently high, exceeding 96% on all diets (*χ*^2^ = 2.02; *p* = 0.57; Figure 2A). Final weight ranged from 1.3 to 1.4 g per PWS individual, regardless of the diet (*Welch's F*_ (3, 57.77)_ = 0.94; *p* = 0.43; Figure 2B). Average weight gain was 1193% (*Welch's F*_ (3, 57.77)_ = 0.94; *p* = 0.42) and the average specific growth rate was 7.2% d^−1^ (*Welch's F*_ (3, 57.23)_ = 1.43; *p* = 0.24), with no significant difference between the PWS groups fed the plant by-products and the control feed (Figure 2C,D). Overall, the PWS individuals in the depectinized apple pomace group showed greater variations in final weight, weight gain, and specific growth rate than the other groups.

### 3.5. Feed Conversion Parameters of BSF-Based Feeds in PWS

Feed conversion parameters and information on the efficiency of conversion of the ingested feed by the PWS are presented in Table 6. Shrimp moisture content varied from 78% to 82% with significant differences among all groups (*F*_(3, 11)_ = 17.02; *p* < 0.001). PWS fed the combined diet with BSF larvae reared on cocoa bean shell had the highest total harvested biomass (FM and DM basis). All PWS fed BSF larvae reared on plant by-products had significantly lower FCR (*Welch's F*_(3, 55.31)_ = 20.25; *p* < 0.001) and significantly higher ECI (*Welch's F*_(3, 56.48)_ = 33.76; *p* < 0.001] values compared to the control feed. ECI and FCR were positively (*r* = 0.63; *p* < 0.001) and negatively (*r* = –0.54; *p* < 0.001) correlated with increasing protein content of BSF larvae, respectively. The fat content of BSF larvae had a comparable effect on ECI (*r* = –0.59; *p* < 0.001) and FCR (*r* = 0.48; *p* < 0.001).

## 4. Discussion

The general goal of this study was to test whether BSF larvae reared on widely available, but underutilized plant by-products may serve as sustainable feeds for PWS aquaculture. Based on randomized controlled feeding trials, we found that (i) the nutritional profiles of the three tested feeds and a grain-based control feed differed in several parameters, (ii) the nutritional profiles of BSF larvae reared on these feeds also differed, (iii) BSF larvae raised on the plant by-products showed significantly lower growth performances than those raised on the control feed, (iv) growth performance parameters of PWS fed BSF larvae reared on plant by-products or the control feed did not show significant differences between groups, and (v) feed conversion parameters of PWS fed BSF larvae reared on the plant by-products were excellent compared to the control group.

### 4.1. Nutritional Profiles of Plant By-Products Differ From Grain-Based Control Feed

Most nutritional profiles of the plant by-products used to rear BSF larvae differed from our grain-based control feed in several parameters (Table 4), particularly in moisture, crude fat, and crude protein contents. While moisture content may not be a critical factor in rearing BSF as feed for PWS, previous studies have shown that fat [10, 11] and, to a lesser extent, protein content [31] may be critical. In fact, the rate at which fish meal-based compounds in PWS feeds can be replaced by BSF-based compounds is highly dependent on the fat content of the BSF larvae [18].

Therefore, it could be expected that a low-fat/high-protein diet for BSF would have a positive effect on growth parameters of PWS. Thus, based of nutritional parameters alone, depectinized apple pomace, and potato peels may be well suited as BSF feeds due to their low fat content, while cocoa bean shells may be suitable due to their high protein content.

However, it should be noted that the plant by-products used in the current study may differ from similar products depending on the place of origin and the plant species or variety used. For example, the protein content of cocoa bean shells can vary worldwide between 10.3% and 27.4% dry weight and the fat content between 1.5% and 8.5% dry weight [20]. Similarly, the fat content of potato peelings may vary between 0.1% and 0.6% of fresh weight (roughly equivalent to 0.6%–2.6% dry weight) and the protein content between 1.2% and 4.4% of fresh weight (roughly equivalent to 7.2%–16.1% dry weight) [32]. These large ranges are not surprising considering, for example, that more than 4000 potato varieties are cultivated [33] and that processing methods for the production of potato peelings may vary.

### 4.2. Nutritional Profiles of BSF Larvae Reared on Plant By-Products Differ From Control Group

As the nutritional profiles of the plant by-products and the control feed differed in several parameters (Section 4.1 and Table 4), we also expected differences in the nutritional profiles of the BSF larvae reared on these feeds. While we observed significant differences between BSF larvae fed plant by-products vs. the control feed for all parameters tested, the crude fat and crude protein contents are particularly noteworthy (Table 5). Crude fat content was lower and crude protein content was higher in all BSF groups reared on plant by-products. For example, BSF larvae fed potato peelings and depectinized apple pomace had a 91% and 84% lower fat content, respectively, than the control group. Similarly, the protein content was 23% and 28% higher in the former and latter treatment groups, respectively, than in the control group.

Considering the nutritional profile of the BSF larvae from this study (Table 5), both potato peelings and apple pomace appear to be the most promising diets for rearing BSF as a sustainable feed for PWS. These low-fat diets may not only reduce the body fat content of BSF, but also adverse health effects in PWS, such as hepatopancreatic damage [17, 19, 34, 35]. Another advantage of the low fat content of BSF larvae reared on depectinized apple pomace and potato peelings is that further processing steps to reduce the fat content of the BSF-based feed, such as defatting [18], might not be necessary. This may save energy, preserve the nutrients contained in the larvae, and increase their attractiveness for direct feeding in aquaculture, although it may still be necessary to cut the larvae into pieces.

Based on the nutritional parameters examined in the current study, both depectinized apple pomace and potato peelings appear to be very suitable plant by-products for rearing BSF as feed for PWS. Even the cocoa bean shells tested showed a slightly more favorable nutritional profile compared to the control feed.

### 4.3. Low Growth Performance of BSF Larvae Fed With Plant By-Products

All nutritional profiles of BSF larvae reared on our plant by-products (Section 4.2 and Table 5) were promising in terms of fat and protein contents. That of BSF reared on cocoa bean shells was even relatively close to that of BSF reared on the grain-based control feed. However, despite these promising nutritional profiles, all growth performance parameters (i.e., larval development time, specific growth rate, and final larval weight) of the BSF reared on plant by-products were significantly inferior to those reared on the control feed (Figure 1). This is particularly evident in the BSF fed depectinized apple pomace and cocoa bean shells, and to a lesser extent in the BSF reared on potato peelings.

These results are broadly consistent with the limited data available in the literature. In a recent study, 12 plant by-products, including apple pomace, were fed to BSF larvae, and their growth performance was recorded [7]. BSF larvae reared on apple pomace showed poor growth rates, resulting in a low final weight (ca. 40 mg). This is in line with our results (final larval weight 27–41 mg; see https://doi.org/10.22029/jlupub-19060) and those of other researchers (e.g., 10–30 mg [11], approximately 40 mg [7], and approximately 80 mg [36]).

To the best of our knowledge, there are no experimental data on growth parameters of BSF fed exclusively with cocoa bean shells. However, in a feeding study with cocoa pods [16], BSF larvae also showed decreasing weight gains and final weights with increasing proportions of cocoa pods in the BSF feed.

With regard to potato peelings, we are not aware of any experimental study in which peels of potato (*Solanum tuberosum*) were used as a complete feed for BSF. However, a study using peels of the sweet potato (*Ipomoea batatas*) showed a significantly reduced weight gain and lower final biomass of BSF compared to BSF fed soy bran [37].

The reasons for the reduced growth performance of BSF fed the above mentioned plant by-products are not well understood and may be by-product specific. A review of the health effects of apple pomace [38] concluded that this by-product is generally safe for human consumption. However, the authors raised concerns about the presence of natural toxins and pesticides when used as animal feed. Of particular relevance is the potent insecticide acetamiprid. In addition, the high content of tannins and anti-nutritional phenolic compounds in apple pomace could also have health implications for BSF [11].

As for cocoa beans, a review [20] raised concerns about heavy metal intake and insecticide contamination. The latter may accumulate particularly in the outer part of the cocoa bean—the cocoa bean shell. The authors also stressed the high concentration of polycyclic aromatic hydrocarbons, which may accumulate during the industrial drying of cocoa beans. They are known toxic chemicals that have been shown to slightly increase the development time of BSF larvae [39].

Finally, potatoes, like many other vegetables, often contain high levels of insecticides, which accumulate particularly in the peel [40]. In addition, potato peelings may have high levels of steroidal alkaloids such as *α*-solanine and *α*-chaconine, which are natural toxins with insecticidal properties [41].

Besides these properties of specific plant by-products potentially affecting the growth characteristics of BSF larvae, there may also be general nutritional limitations. For example, studies have shown that fruit and vegetable by-products are low in essential amino acids [42]. This may contribute to a prolonged developmental time of BSF larvae reared on plant by-products [7]. Therefore, supplementation with specific essential amino acids that are lacking in plant by-products may be a key to optimizing plant by-products as feed for BSF larvae and increasing growth performance of BSF.

### 4.4. No Difference in Growth Performance of PWS Fed With BSF Larvae Reared on Plant By-Products

Our analyses of the nutritional profiles of the BSF larvae reared on plant by-products showed significant differences between these feeds and the grain-based control feed (Section 4.2). In addition, our analyses of the growth performance of BSF larvae reared on these by-products showed significantly delayed developmental processes and reduced final weights (Section 4.3). Therefore, we also expected lower growth performance in our PWS reared on by-product-based combined diets (i.e., 50% conventional fish meal-based compound feed and 50% BSF larvae reared on by-products) compared to our control feed (i.e., 50% conventional fish meal-based compound feed and 50% BSF larvae reared on a grain-based feed).

However, our randomized controlled trial showed no significant differences in growth parameters between the PWS treatment groups or between the treatment and control groups (Figure 2). Survival rates of PWS were greater than 96% for all feeds tested (Figure 2A). In addition, final weights, weight gains, and specific growth rates (Figure 2) were very similar. This suggests that potential nutritional deficiencies or contaminants and toxins of the plant by-products may affect the BSF (Section 4.3), but not the PWS fed these BSF larvae. We can only speculate as to the reasons for these findings. Possibly, BSF larvae do not bioaccumulate some of these contaminants and toxins, but rather degrade them, as recently proposed for polycyclic aromatic hydrocarbons [39] and for the toxic polyphenolic compound gossypol [14]. Furthermore, BSF may partially compensate for the deficiency of some essential amino acids in plant by-products [43]. Accordingly, the authors found that BSF fed different vegetable by-products all had essential amino acid compositions close to the FAO nutritional requirements, at least for humans.

These results may have important economic implications for commercial shrimp aquaculture, as BSF larvae reared on plant by-products may not require defatting or supplementation of essential amino acids to be used as PWS feeds.

### 4.5. Good Feed Conversion Parameters in PWS Fed With BSF Larvae Reared on Plant By-Products

The results of our feed conversion analyses are given in Table 6 and are rather unexpected. Our experimental feeds based on BSF reared on plant by-products showed excellent FCRs, ranging from 0.8 (BSF fed depectinized apple pomace) to 1.0 (BSF fed cocoa bean shells). These values were also better than the PWS control group, which had an FCR of 1.4.

In well-managed PWS farms, FCR ranges from 1.3 to 1.5, and in average PWS farms from 1.6 to 1.8. In poorly managed farms, the FCR can be as high as 2.5 (reviewed in [44]). Furthermore, in experimental studies where defatted BSF meal was used to rear PWS, FCR ranged from 1.1 to 1.4 [18, 19]. FCR also depends on the age of the PWS, with young individuals or larvae often having better values, which can be as low as 1.1 [31].

The FCR determined in our study has important implications, as it shows that the low specific growth rates of BSF fed plant by-products (Figure 1 and Section 4.3) may be fully (potato peelings) or partially (cocoa bean shells and depectinized apple pomace) compensated for by the better FCR of the PWS reared on these BSF. The excellent FCR in our treatment groups may also help to reduce the amount of feed required for BSF-based aquaculture, with potentially positive side effects on water quality and nutrient pollution.

The reasons for these exceptional FCRs are not known to us. However, our empirical observations suggest that the BSF larvae reared on plant by-products provide better feeding stimuli to the PWS than those reared on the control feed. This may be due to the often higher protein and lower fat contents of the BSF reared on plant by-products. In addition, the excellent FCR could also be related to differences in the composition of macro- and micronutrients, which vary depending on the feed and rearing conditions [45].

Our finding of outstanding FCR could also be of economic importance for commercial shrimp farming, as it suggests that some as yet unspecified characteristics of BSF reared on plant by-products could significantly improve feed conversion parameters.

### 4.6. Limitations and Outlook

In our study, we used BSF-based feeds to investigate nutritional and growth-performance parameters in PWS. Both the treatment and control feeds consisted of 50% fresh BSF larvae. Therefore, our results of similar growth performance and excellent FCR of PWS fed with BSF larvae reared on plant by-products may be primarily applicable to shrimp aquaculture systems, where BSF larvae are fed directly to the PWS.

In large-scale aquaculture facilities, PWS are typically reared on extruded feed pellets, which would require processing of BSF. On the one hand, these processing steps may alter the macro- and micronutritional profiles of the BSF and lead to a reduction in feeding stimuli. On the other hand, previous studies have suggested that processing of BSF larvae (e.g., drying, cuticle removal) may also increase nutrient availability and/or acceptability of the BSF [45].

Therefore, we suggest future studies using pelleted feed with meal of the BSF, which were reared on plant by-products. We also recommend controlled trials aimed at improving the growth parameters of BSF fed with plant by-products, for example by adding essential amino acids. The fact that the nutritional profiles of BSF reared on plant by-products are very promising in terms of protein and fat contents would also be of interest for future research. In particular, the low fat content could make it possible to replace more than 50% of the fish meal-based components in PWS feeds with BSF-based compounds. Finally, the excellent FCR found in PWS fed with BSF reared on plant by-products call for future studies on the drivers of these improved parameters, for example through specific micro- and macronutritional analyses.

## 5. Conclusions

Our study shows that the nutrient profiles of three plant by-products, selected for their low economic value and high availability (i.e., cocoa bean shells, depectinized apple pomace, and potato peelings), differ in several parameters, particularly in crude fat and crude protein contents.

The nutritional profiles of BSF larvae reared on these three experimental feeds also differed from each other and from the grain-based control feed, particularly in crude fat, crude protein, and ash contents. Based on these nutritional parameters, both depectinized apple pomace and potato peelings appear to be very suitable plant by-products for rearing BSF as a feed for PWS, possibly eliminating the need for defatting the BSF.

However, all BSF reared on the plant by-products showed significantly lower growth performances than those reared on the control feed. The reasons for this are largely unknown. Though, by-product-specific parameters (e.g., specific toxins and insecticides) and general parameters (e.g., the low content of some essential amino acids in plant by-products) may play a role.

In contrast to the varying growth performances of the BSF, PWS fed with BSF larvae reared on plant by-products or the control feed showed no significant differences in growth parameters. The reasons for the consistent growth performance of the PWS also require future investigation. It is possible that BSF do not bioaccumulate some of the contaminants and toxins contained in the plant by-products. BSF may also partially compensate for the deficiency of some essential amino acids in the plant by-products.

Finally, we found excellent FCR for our PWS fed with BSF larvae reared on plant by-products, well below published values for commercial shrimp farming and experimental studies with BSF. These excellent FCR may fully (potato peelings) or partially (cocoa bean shells and depectinized apple pomace) compensate for the lower growth performance of the BSF reared on these by-products. The reasons for these superior FCR are still unknown, but the high feeding stimuli provided by our experimental feeds and specific micro- and macronutritional profiles may play a role.

Our results could be of great economic importance for future shrimp aquaculture, confirming that BSF may be an important link in the conversion of low-cost plant by-products into high-quality and valuable protein sources for sustainable shrimp farming in a circular economy.

To further facilitate the industrial application of plant by-products for insect and shrimp production, we recommend several future research directions using BSF reared on plant by-products: (i) studies with BSF meal, (ii) studies to improve growth parameters in BSF, (iii) studies with BSF replacement rates in shrimp feeds >50%, and (iv) studies to decipher the factors responsible for the excellent FCR in PWS.

## Figures and Tables

**Table 1:** Characteristics of the black soldier flies (BSFs) and Pacific white shrimp (PWS) studied.

Characteristic	BSF	PWS
Species name	Black soldier fly, *Hermetia illucens* Linnaeus, 1758	Pacific white shrimp, *Penaeus vannamei* Boone, 1931
Origin	Bio.S Biogas (Grimma, Germany)	Suburban Seafood Germany UG (Nebelschütz, Germany)
Genetic strain	For DNA barcoding information on the strain used see GenBank accession #PQ187607 and PQ187608	For DNA barcoding information on the strain used see GenBank accession #PQ465998 and PQ465999
Life stage	Fifth larval instar	Juvenile specimens (7 weeks old)
Weight	36–253 mg	0.10 ± 0.01 g
Length	0.5–2.5 cm	2.4 ± 0.6 cm

**Table 2:** Experimental conditions of the black soldier flies (BSFs) and the Pacific white shrimp (PWS).

Characteristic	BSF	PWS
Duration of trials	17–82 days (≥50% prepupae)	35 days
Rearing system	Small-scale indoor rearing	Indoor clear water recirculation system
Rearing container	19.5 cm × 16.5 cm × 9.5 cm polypropylene boxes, maximum filling capacity of 2 L, 12 boxes in total	35 cm × 21 cm × 20 cm plastic tanks, filling capacity of 9.2 L, each divided in two chambers by a plastic wall, 56 tanks in total
Replications	*n* = 3	*n* = 28
Water filtration system	N/A	Vlies Dreambox 3.0 with fleece filter and protein skimmer (Royal Exclusiv), and a flow-through biofilter (Aqualight) with a sponge media
Photoperiod	24 h dark	Blue LED light (ca. 12 h light/12 h dark)
Stocking density	150 mg eggs per box (~6000 eggs)	1 postlarva per chamber
Feed	Fed *ad libitum* (typically at 48-h intervals) with CBS, DAP, PP, and CF	Four times daily at 6:00 am (compound feed), 9:30 am (BSF larvae), 4:00 pm (BSF larvae) and 10:00 pm (compound feed) to a visual satiation each time
Method of processing	Cleaned and stored in a freezer (–20°C) until use	PWS were weighed, measured, and photographed at the start and end of the trials. After 35 days, the shrimp were euthanized, placed in bags, and stored in a freezer (–20°C)

Abbreviations: CBS, cocoa bean shells; CF, control feed; DAP, depectinized apple pomace; PP, potato peelings.

**Table 3:** Water parameters for the PWS preexperimental and experimental periods.

Water parameter	Value
Salinity (ppt)	24.3 ± 0.5
Temperature (°C)	27.7 ± 0.2
pH	7.8 ± 0.1
Dissolved oxygen (%)	97.5 ± 2.1
Nitrite (mg L^−1^)	0.1 ± 0.2*⁣*^*∗*^
Nitrate (mg L^−1^)	24.3 ± 9.5

*Note*: Data represent mean ± SD.

*⁣* ^*∗*^Only found in the first week, afterward 0.0 mg L^−1^.

**Table 4:** Nutritional profiles of three plant by-products (cocoa bean shells, depectinized apple pomace, potato peelings) and a grain-based control feed.

Parameter	BSF feed substrates			
	Cocoa bean shells (*n* = 3)	Depectinized apple pomace (*n* = 3)	Potato peelings (*n* = 3)	Control feed (*n* = 3)
Moisture (% FM)	6.5 ± 1.0^b^	9.5 ± 0.5^b^	81.9 ± 3.2^a^	6.7 ± 0.3^c^
Crude ash (% DM)	6.4 ± 0.0^c^	3.6 ± 0.1^d^	2.8 ± 0.1^b^	12.9 ± 0.9^a^
Crude fat (% DM)	30.1 ± 0.7^c^	3.2 ± 0.0^d^	0.4 ± 0.0^b^	2.5 ± 0.0^a^
Crude protein (% DM)	20.3 ± 0.0^c^	8.0 ± 0.1^b^	7.5 ± 0.8^b^	18.2 ± 0.4^a,^*⁣*^*∗*^
Total nitrogen (% DM)	3.3 ± 0.0^c^	1.3 ± 0.0^b^	1.2 ± 0.1^b^	2.9 ± 0.1^a,^*⁣*^*∗*^

*Note*: Different letters (a–d) indicate statistically significant differences (*p* < 0.05). Data represent mean ± SD.

Abbreviations: DM, dry matter; FM, fresh matter.

*⁣* ^*∗*^ *n* = 4 with data taken from [14].

**Table 5:** Nutritional profiles of black soldier fly (BSF) larvae reared on three plant by-products (cocoa bean shells, depectinized apple pomace, potato peelings) and a grain-based control feed.

Parameters	BSF larvae reared on			
	Cocoa bean shells (*n* = 3)	Depectinized apple pomace (*n* = 3)	Potato peelings (*n* = 3)	Control feed (*n* = 3)
Moisture (% FM)	70.1 ± 1.1^c^	81.7 ± 0.5^d^	79.2 ± 0.2^b^	64.3 ± 0.0^a^
Crude ash (% DM)	5.4 ± 0.2^a^	9.0 ± 0.3^c^	12.3 ± 0.7^b^	4.9 ± 0.2^a^
Crude fat (% DM)	28.9 ± 0.8^c^	5.3 ± 0.4^b^	3.1 ± 0.4^b^	33.9 ± 1.1^a^
Crude protein (% DM)	46.9 ± 0.2^c^	52.1 ± 0.3^b^	50.2 ± 0.9^b^	40.7 ± 1.3^a,^*⁣*^*∗*^
Total nitrogen (% DM)	7.5 ± 0.0^c^	8.3 ± 0.0^b^	8.0 ± 0.1^b^	6.5 ± 0.2^a,^*⁣*^*∗*^

*Note*: Different letters (a–d) indicate statistically significant differences (*p* < 0.05). Data represent mean ± SD.

Abbreviations: DM, dry matter; FM, fresh matter.

*⁣* ^*∗*^ *n* = 5 with data taken from [14].

**Table 6:** Feed conversion parameters of PWS fed a combined diet consisting of a compound feed as well as BSF larvae reared on plant by-products or a grain-based control feed.

Parameters	PWS feeding group			
	Cocoa bean shells	Depectinized apple pomace	Potato peelings	Control feed
Initial weight (g FM), *n* = 112	0.1 ± 0.0			
Moisture of shrimp (% FM), *n* = 3	81.9 ± 0.9^c^	80.7 ± 0.3^ac^	78.4 ± 0.3^b^	80.3 ± 0.3^a^
Total harvested biomass (g FM), *n* = 28	40.5	37.3	36.7	37.2
Total harvested biomass (g DM), *n* = 28	7.3	7.2	7.9	7.3
Feed conversion ratio, *n* = 28	1.0 ± 0.2^bd^	0.9 ± 0.5^b^	0.8 ± 0.1^bc^	1.4 ± 0.3^a^
Efficiency of conversion of ingested feed, *n* = 28	1.0 ± 0.2^c^	1.4 ± 0.5^b^	1.3 ± 0.3^b^	0.8 ± 0.2^a^

*Note*: Data are mean ± SD. Different letters (a–d) within a row indicate statistically significant differences (*p* < 0.05; one-way ANOVA or Welch's ANOVA).

Abbreviations: DM, dry matter; FM, fresh matter.

## Data Availability

The data that support the findings of this study are openly available in JLUpub at https://doi.org/10.22029/jlupub-19060, reference number 19703.
